# Supplementary material for: Altered Expression of BCRP Impacts Fetal Accumulation of Rosuvastatin in a Rat Model of Preeclampsia
Source: Pharmaceutics. 2024 Jun 30;16(7):884. doi: 10.3390/pharmaceutics16070884 (PMC11279450; doi:10.3390/pharmaceutics16070884)
Supplement: Supplementary file 1 [file pharmaceutics-16-00884-s001.zip › pharmaceutics-3054222-supplementary.pdf]

Supplementary Materials

A B

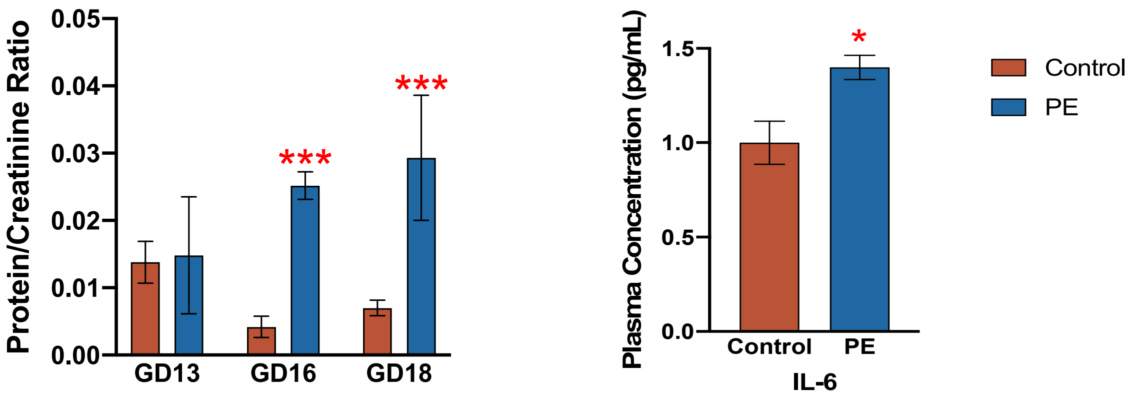

Figure S1. Confirmation of PE characteristics in the immunological rat model of PE. **A.** Urinary Total Protein:Creatinine Ratios. Urine was collected from PE and control rats (n = 15–16/group) at baseline on GD13, and after induction of PE on GD16, and GD18. **B.** IL-6 Serum Concentrations. Protein levels of IL-6 were measured in serum obtained from PE and control dams by ELISA. Data is presented as mean ± S.D. (n = 15–16/group). Significance was determined using Student's unpaired t-test (* $p < 0.05$, ** $p < 0.01$, *** $p < 0.001$).

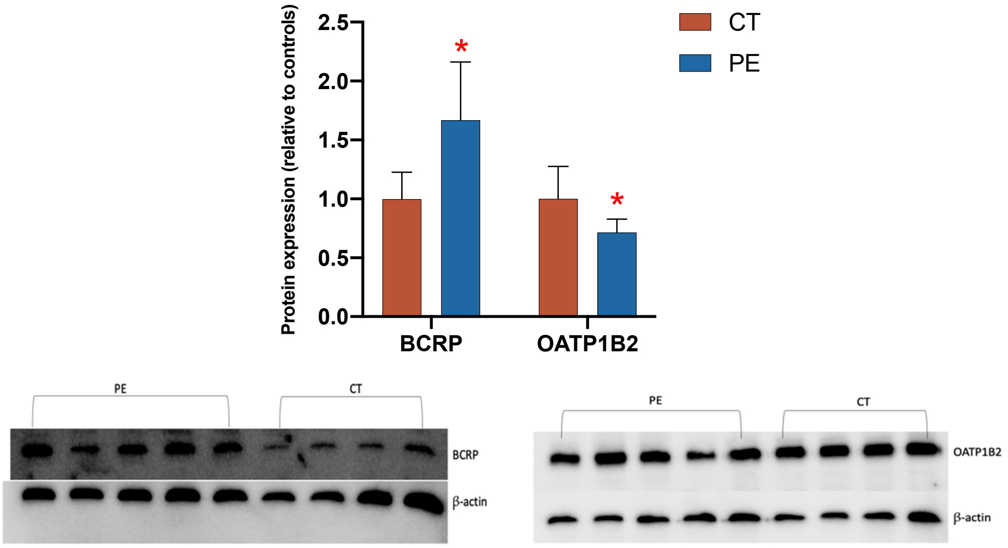

Figure S2. Hepatic protein expression of Bcrp and Oatp1b2. Protein expression in control and PE dams was measured by western blot assay and normalized to β-actin level. Data is presented as mean ± S.D. (n = 15–16/group). Significance was determined using Student's unpaired t-test (* $p < 0.05$). Representative blots are shown below the graph.

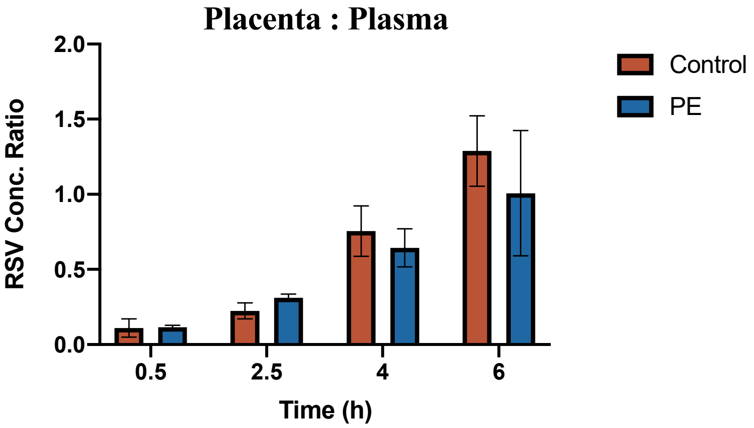

Figure S3. Rosuvastatin placenta:plasma concentration ratio in control rats and PE rats. The concentrations of RSV in the placenta (ng/g) was normalized to the concentration of RSV in maternal plasma (ng/ml) at the same time point and are shown as placenta:plasma concentration ratio (n = 3–4/group). RSV concentrations were determined as described in methods and are presented as mean ± S.D. Significance was determined using Student's unpaired t-test (* $p < 0.05$; ** $p < 0.01$).
